# Remdesivir-bound and ligand-free simulations reveal the probable mechanism of inhibiting the RNA dependent RNA polymerase of severe acute respiratory syndrome coronavirus 2†

**DOI:** 10.1039/d0ra04743k

**Published:** 2020-07-17

**Authors:** Shruti Koulgi, Vinod Jani, Mallikarjunachari V. N. Uppuladinne, Uddhavesh Sonavane, Rajendra Joshi

**Affiliations:** High Performance Computing Medical and Bioinformatics Applications Group, Centre for Development of Advanced Computing (C-DAC) Panchvati, Pashan Pune India rajendra@cdac.in

## Abstract

The efforts towards developing a potential drug against the current global pandemic, COVID-19, have increased in the past few months. Drug development strategies to target the RNA dependent RNA polymerase (RdRP) of Severe Acute Respiratory Syndrome Coronavirus 2 (SARS-CoV-2) are being tried worldwide. The gene encoding this protein, is known to be conserved amongst positive strand RNA viruses. This enables an avenue to repurpose the drugs designed against earlier reported inhibitors of RdRP. One such strong inhibitor is remdesivir which has been used against EBOLA infections. The binding of remdesivir to RdRP of SARS-CoV-2 has been studied using the classical molecular dynamics and ensemble docking approach. A comparative study of the simulations of RdRP in the apo and remdesivir-bound form revealed blocking of the template entry site in the presence of remdesivir. The conformation changes leading to this event were captured through principal component analysis. The conformational and thermodynamic parameters supported the experimental information available on the involvement of crucial arginine, serine and aspartate residues belonging to the conserved motifs in RdRP functioning. The catalytic site comprising of SER 759, ASP 760, and ASP 761 (SDD) was observed to form strong contacts with remdesivir. The significantly strong interactions of these residues with remdesivir may infer the latter's binding similar to the normal nucleotides thereby remaining unidentified by the exonuclease activity of RdRP. The ensemble docking of remdesivir too, comprehended the involvement of similar residues in interaction with the inhibitor. This information on crucial interactions between conserved residues of RdRP with remdesivir through *in silico* approaches may be useful in designing inhibitors.

## Abstract 2

Conformational changes in the remdesivir-bound RdRP leading to its inhibition.

## Graphical abstract

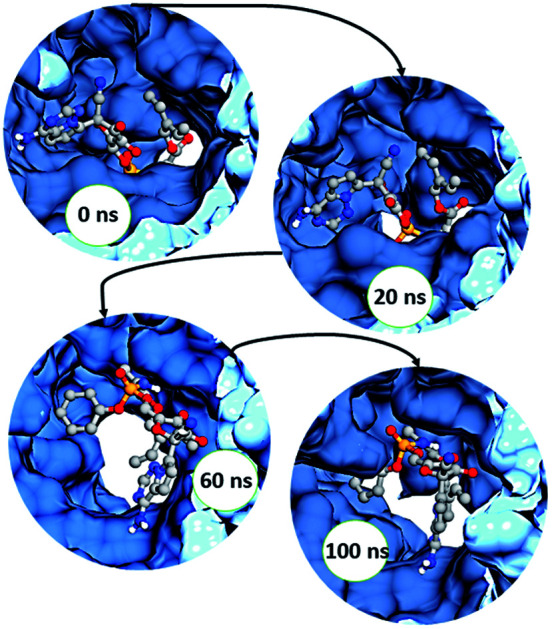

## Introduction

**Fig. 1:**
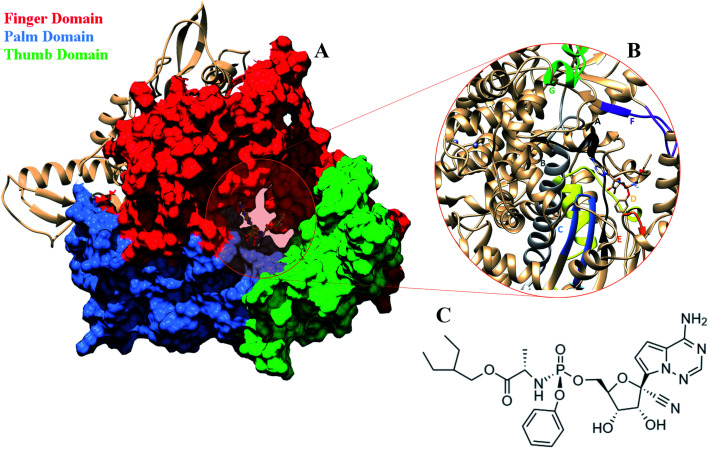
(A) RdRP structure in the cupped righthand form bound to remdesivir (RDV) with the three subdomains finger (red) (residues 398–581 and 621–679), palm (cornflower blue) (residues 582–627) and thumb (green) (residues 688–815). (B) The conserved motifs (A–G) surrounding the inhibitor binding cavity. (C) 2-d representation of the inhibitor remdesivir (RDV), Sp isomer.

COVID-19 that emerged as global pandemic is spreading in an uncontrollable manner, in spite of the safety norms and the therapeutics that have been employed to overcome the disease. Researchers across the globe have been investigating strategies that would help to stop the viral duplication, thereby reducing its spread.^1^ COVID-19 is known to be caused by the SARS-CoV-2 virus which belongs to the family of coronaviruses.^2^ The viruses from this family are known to have largest RNA genomes. SARS-CoV-2 has a ∼30 K bases long RNA genome which codes for about 29 proteins.^3^ These have been categorized into pp1a, pp1b, structural proteins and accessory proteins. The ORF1a and ORF1b code for two long polypeptide chains namely, pp1a and pp1b which are cleaved by the viral proteases and lead to the generation of 16 non-structural proteins (nsp).^2,3^ These proteins are named as nsp1-16 each of these performing their individual viral functions. Amongst these nsp, the nsp12, is the RNA dependent RNA polymerase (RdRP) which is responsible for viral duplication.^4^ Duplication being the primary function for any virus to survive, the gene that codes for this protein are known to be the most conserved.^5–9^ Sequence similarity studies suggests that it shows maximum identity with the RdRP of SARS-CoV virus, which was known to affect the humans in and around the year 2003.^10^ The sequence of SARS-CoV-2 RdRP shares 96.3% similarity with the RdRP of SARS-CoV.^11^ Owing to its function, it has been a strong candidate to develop inhibitors against, as blocking this protein would lead to reduction in the spread of the virus. The cryoEM structure of the SARS-CoV-2 was elucidated in the first week of May 2020 with PDB ID 7BV1.^12^ The RdRP structurally resembles to that of a cupped right hand.^12^ The polymerase domain is divided into three subdomains, namely, finger, palm and thumb subdomain (Fig. 1A). The finger subdomain consists of the residues 398–581 and 621–679. The palm subdomain is the largest among the three domains and comprises of the residues 582–627 and 688–815. The thumb subdomain is the smallest of three and consists of residues 816–919. A nidovirus RdRP-associated nucleotidyltransferase domain (NiRAN) is present within the residue range 117–250. The RdRP being one of the most conserved proteins amongst the positive strand RNA viruses, consists of seven conserved motifs (A–G) which consists of crucial residues that are required by the RdRP to perform its function.^8,12^Fig. 1B shows the location of these motifs. The motif A (residues 613–626), B (residues 675–710), C (residues 753–767) and D (residues 772–796) are a part of the palm subdomain. The catalytic site SDD of RdRP lies in the motif C, SER 759, ASP 760 and ASP 761 are involved in primer binding for RdRP functioning.^8,12^ The motif E (residues 811–821) contains another primer binding site CSQ, formed by CYS 813, SER 814 and GLN 815. The motif F (residues 544–555) consists of LYS 545 ARG 555 which is known to bind to inhibitors. The motif G (residues 500–514) consists of LYS 500, SER 501 and ASN 507 which are known to bind to the template residues. Most of the inhibitors designed against RdRP are known to interact with the residues of these conserved motifs and lead to loss in functionality of this protein.^13^ In the current situation, the increase in number of cases of COVID-19 worldwide demands development of faster therapeutics against it. The knowledge of homology and conservation of protein sequence along these virus families helps in deducing the potential inhibitors against COVID-19. Drug repurposing using previously known RdRP inhibitors would help in developing faster solutions.^14^ A known class of RdRP inhibitors along the coronavirus consists of nucleoside analogues, that interfere with activity of RdRP. There have been *in vitro* as well as *in silico* studies reporting the activity of the nucleosides namely, remdesivir, favipiravir, sofosbuvir, galidesivir and tenofir against RdRP.^15–17^ Each of the nucleoside analogues pose as potential candidates for antiviral treatment against COVID-19.^18–20^ The nucleoside analogue remdesivir (referred as RDV), has been previously used as a treatment against EBOLA virus (Fig. 1C).^21,22^ It is also known to inhibit the RdRP of the MERS coronavirus.^23^ Experiments suggest that remdesivir has been capable of inhibiting the RdRP of the current pandemic causing SARS-CoV-2 with high potency.^24,25^ The potential use of remdesivir as a combination therapy with chloroquinone and azithromycin is known to explored.^26^ Selective treatment guidelines have allowed administration of RDV as a drug in critically ill patients.^27^ The US National Institute of Allergy and Infectious Diseases (NIAID) had announced the use of RDV is enabling the SARS-CoV-2 infected patients to recover faster.^28,29^ The major concern in case of COVID-19 lies in decreasing the mortality rate and speeding up the recovery rate of the infected patients. To achieve this, it has become important to gain approval on the usage of drugs like RDV to be administered in COVID-19 patients. A recent development in structural biology has revealed the inhibitory effect of RDV captured through cryo-Electron Microscopy.^12^ A nsp12–nsp7–nsp8 complex bound to primer and RDV was published in the first week on May 2020. The structure has been deposited in the PDB with ID 7BV2.^12^ A correction over this structure was also reported in June 2020 which had an addition at the C-terminal end and the removal of Mg^2+^ ion.^30^ These structures have enabled researchers to develop inhibitors against SARS-CoV-2 RdRP by taking into account the interactions observed between RdRP and RDV and understanding their role in the inhibitory effect. The structure has revealed the role of LYS 545, LEU 759 and SER 814 by forming strong non-covalent interactions between the drug and the target.^12^ However, there may be many other interactions too that would be possibly captured only on studying the dynamics of the RdRP in the presence of RDV. Studies have been reported wherein simulation studies have helped in understanding the mechanism of remdesivir interaction with the RNA template in order to block the RdRP.^31–33^ The work reported in the current paper shows the interactions of remdesivir with the RdRP residues and the changes the enzyme undergoes on binding to this inhibitor through multiple molecular dynamics simulations.

Classical molecular dynamics simulations were performed for the SARS-CoV-2 RdRP in the apo form and in the RDV-bound form. Three replicates of 50 ns each were performed for the apo form of RdRP (referred as RdRP–Apo). Similarly, three replicates of simulations were performed for the RDV-bound RdRP (referred as RdRP–RDV), wherein two replicates were simulated for 50 ns each and the third was extended to complete 100 ns. Hence, an overall simulation data of 350 ns was analyzed and has been reported. When the work was initiated, no structure of SARS-CoV-2 RdRP was available. Hence, the homology model was developed using SWISS-MODEL and SARS-CoV RdRP with PDB ID 6NUR was considered as the template.^34,35^ Owing to high percentage of sequence similarity between SARS-CoV and SARS-CoV-2, a high-quality homology model was generated and was used for the simulation studies. The RdRP–RDV structure was obtained by rigid docking of RDV (PubChem CID: 121304016) on the RdRP model followed by molecular dynamics of RdRP–Apo and RdRP–RDV systems. A comparative analysis of the RdRP–Apo and RdRP–RDV simulations was performed based on the conformational parameters like Root Mean Square Deviation (RMSD) and Root Mean Square Fluctuation (RMSF). Principal component analysis (PCA) was performed on both the simulations, which helped in identifying significant conformational variations in RdRP–Apo and RdRP–RDV. Further, the RdRP–RDV simulations were extensively analyzed to associate the conformational changes with the inhibitory effect of RDV. Conformational parameters like hydrogen bond and native contact formation were calculated to spot the involvement of residues of RdRP in interacting with RDV. The MM-GBSA free energy analysis was also performed in order to comprehend the observations with thermodynamic parameters. All the analysis led to identification of residues that were significantly involved in interacting with RDV. Most of these residues belonged to the conserved motifs (A–G) or were a part of the catalytic site important for initiation of primer binding. An ensemble docking of RDV was performed on the ensemble representatives obtained from the RdRP–Apo simulations. The RDV interactions observed in the ensemble docked conformations helped to encompass the results obtained through simulations. The entire *in silico* approach presented in this article aims to identify the different types of interactions that lead to the inhibitory effect of RDV on RdRP.

## Methodology

### Model generation

The structure for SARS-CoV-2 RdRP was not present at the time this work was started. Hence, 3D co-ordinates for the RdRP model were obtained from the model of SARS-CoV-2 built using SWISS-MODEL. The template used here was the RdRP of SARS-CoV from the PDB ID 6NUR.^34^ This template was present in complex with nsp7 and nsp8. However, the N-terminal was missing for the SARS-CoV-2 RdRP model generated through homology modelling. The predicted model consists of residues 117–919 with the presence of two Zinc ions in coordination with CYS and HIS residues. One of the zinc ions was present in a tetrahedral co-ordination complex formed by CYS 301, CYS 306, CYS 310 and HIS 295 and the other in co-ordination with CYS 487, CYS 645, CYS 646 and HIS 642. During the course of this work, the structure for SARS-CoV-2 RdRP was revealed through cryoEM studies.^12^ The structures with PDB ID 7BV1 and 7BV2 was elucidated, the former one being the apo form in complex with nsp7 and nsp8.^12^ The latter one being primer and RDV-bound and in complex with nsp7 and nsp8. In order to check the resemblance of the RdRP model with newly discovered structure of SARS-CoV-2 RdRP, the backbone RMSD for the residues 117–919 was calculated. Fig. S1† shows the superimposition of the RdRP model used in this study (pink) and the PDB ID 7BV2 (blue). The backbone RMSD was observed to be 0.744 Å, which suggests that the predicted RdRP model was in well agreement with the experimental structure. The coordinates for the RDV molecule used for the binding studies were obtained from PubChem with CID 121304016/Drug Bank Accession Number DB14761. The molecule used is the Sp isomer of RDV, which is known to have more therapeutic potential as compared to its other isomer.^22^ The binding studies of RDV performed using the RdRP model have been reported in this article.

Two approaches have been carried out to understand the binding of the RDV to RdRP. The first approach involves rigid docking of the RDV to the RdRP model followed by molecular dynamics simulations. The second approach was ensemble docking of RDV to the ensemble representatives of RdRP generated through molecular dynamics simulations of the RdRP–Apo.

### Molecular docking

The 3D conformation of RDV-bound RdRP was obtained from rigid docking of the RDV to the RdRP model. The receptor parameters were generated using UCSF Chimera.^36^ The AMBER14SB force field was used for parameterization. This was followed by identification of the active site using the *sphgen* module of DOCK 6.^37^ Spheres were generated on the surface of the receptor molecule and the cluster with most populated sphere was considered as the active site. This was followed by generation of grid over the active site, in order to effectively accommodate the ligand molecule. This was performed by the *grid* module of DOCK 6.^37^ The last step was docking of the ligand, RDV to the prepared receptor, RdRP. Standard parameters were used for the RDV molecule.

### Molecular dynamics

Classical molecular dynamics simulations were performed for the RdRP–Apo and RdRP–RDV systems. The AMBER 16 simulation package was used for performing the simulations.^38^ The AMBER14SB force field was used for generating the parameters for the RdRP. The antechamber module of AMBERTOOLS along with the GAFF force field was used to generate the parameters for RDV.^39,40^ The zinc co-ordination complexes were treated using the Zinc Amber Force Field (ZAFF).^41^ The entire simulation system was neutralized by addition of sodium ions. The protein and protein–ligand system were solvated using the TIP3P water model. The minimization was performed in two stages, 20 000 steps using the steepest descent method followed by 10 000 steps of the conjugate gradient method. Minimization was followed by temperature ramping. The solvent alone was first heated gradually to attain a temperature of 300 K, by adding force restraints to the solute. This was followed by gradual heating of the entire simulation system to 300 K. The Langevin thermostat was used to maintained the temperature at 300 K. The SHAKE algorithm was used for handling the hydrogen restraints. NPT equilibration was performed for 1 ns at temperature of 300 K and 1 atm pressure. This was followed by production run of 50 ns for RdRP–Apo and RdRP–RDV. Three replicates of 50 ns production run were performed for RdRP–Apo system. Two replicates of 50 ns and a single run that was extended up to 100 ns of production MD for RdRP–RDV system.

### Ensemble generation and docking

The simulation data of the RdRP–Apo was used for generating representative of difference conformational ensembles of RdRP. The simulation data was clustered method using the density-based clustering methods in order to generate the ensemble representatives. The density-based clustering method was achieved by using the *dbscan* option available through the *cpptraj* module of AmberTools 17.^42,43^ An RMSD cut-off of 1.7 Å was used, which resulted in the formation of five clusters. The cluster centers of these five clusters were considered as the ensemble representatives are were used for docking of RDV. The RMSD of these ensemble representatives against the RdRP of PDB ID 7BV2 was calculated. RMSD values of 2.032 Å, 2.164 Å, 2.014 Å, 2.9 Å and 1.68 Å were obtained for the five clusters. These ensemble representatives were docked with RDV using the same molecular docking protocol as described in the “Molecular Docking Section” in Methods.

## Results and discussion

### Comparison between RdRP–Apo and RdRP–RDV

**Fig. 2:**
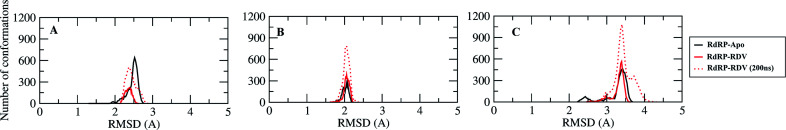
RMSD of the three RdRP subdomains, finger (A), palm (B) and thumb (C) against the RdRP modelled structure.

**Fig. 3:**
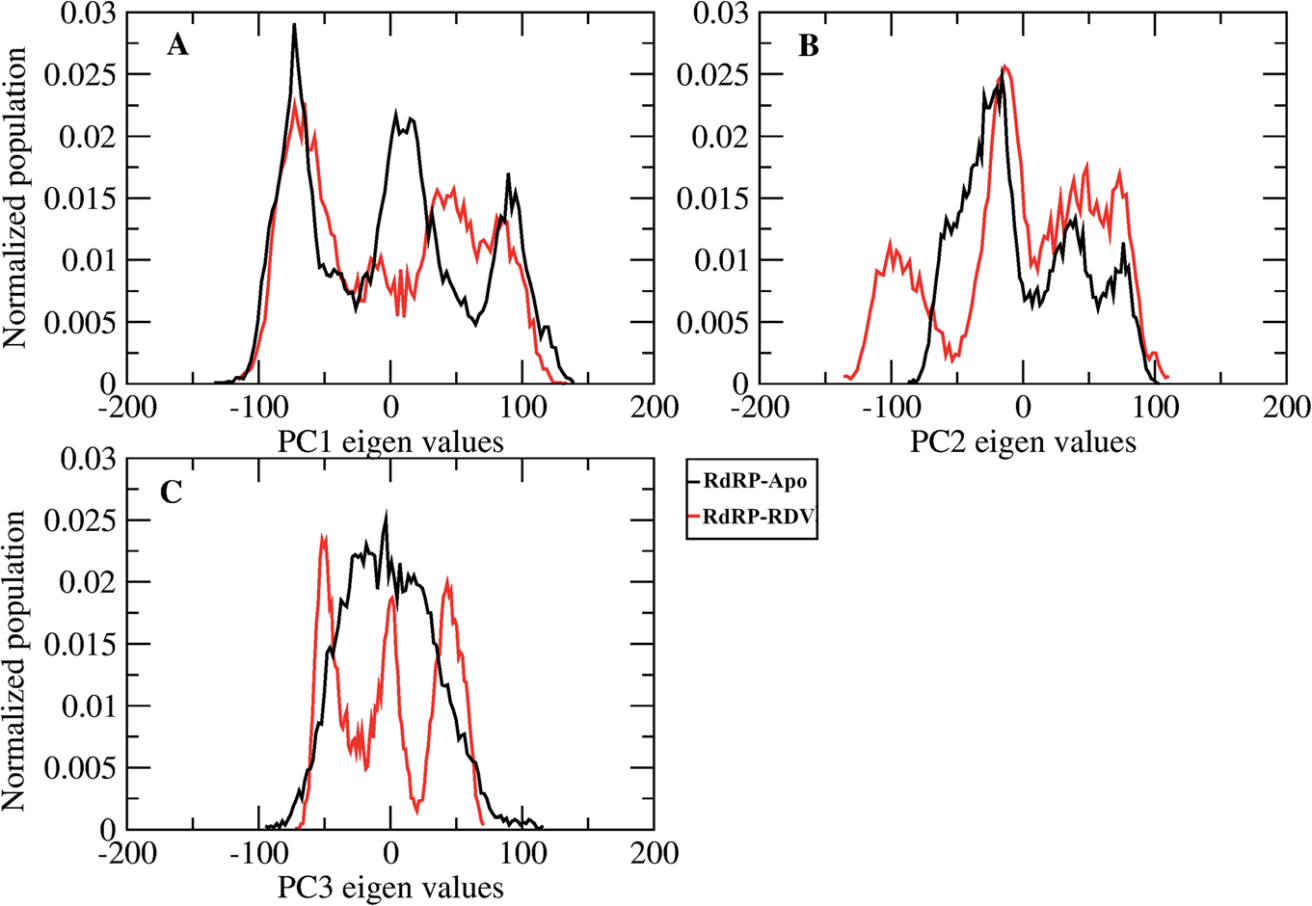
Population distribution of the conformations along the first three principal components, PC1 (A), PC2 (B) and PC3 (C).

**Fig. 4:**
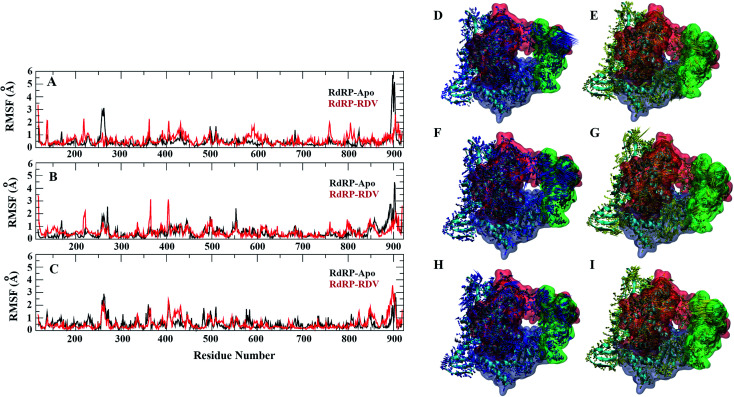
Residue-wise fluctuation captured by PC1, 2 and 3 (A, B and C). Directionality of the fluctuations captured by PC1, PC2 and PC3 for apo (D, F and H) and rdz-bound (E, G and I) simulations.

A comparative analysis of the conformational changes occurring in the RdRP protein when present in the apo form and remdesivir-bound form were performed. The RMSD and root mean square fluctuation (RMSF) of the RdRP residues were calculated using the *cpptraj* module of AmberTools 17.^43^ As the simulations were performed in three replicates with conformations captured at every 10 ps, the RMSD and RMSF values at every 10^th^ ps were averaged over the three replicates and used for comparison. However, as one of the replicates for remdesivir-bound simulations was performed till 100 ns, to maintain uniformity a histogram plot depicting the number of conformations obtained at different RMSD values has been shown in Fig. S2A.† The observations for 150 ns of RdRP–Apo and RdRP–RDV have been shown in black and red, whereas, the dotted red line depicts the RdRP–RDV values with the additional 50 ns. The RdRP protein showed major population having RMSD values between 2.5 to 3 Å. A single fine peak was obtained for RdRP–Apo as compared to multiple short peaks for the RdRP–RDV. However, the extended simulation of RdRP–RDV sampled conformations that populated well to obtain a single fine peak. Majority of RdRP conformations obtained through the simulations were observed to deviate from the start model by 2.7 to 3 Å in case of apo as well as RDV-bound form. Based on the RMSD values the overall conformational dynamics of the RdRP appeared to be similar in both, apo and RDV-bound form. Hence, the RMSD was calculated for the three domains namely, finger, palm, and thumb. Fig. 2A, B and C shows the histogram plots for the RMSD values of finger, palm and thumb subdomains respectively. Similar to the overall structure of RdRP, the finger and the thumb subdomain showed similar RMSD values for the RdRP–Apo and RdRP–RDV simulations. However, it was observed that for the palm subdomain two conformations were populated in terms of the RMSD values for the RdRP–Apo simulations as compared to one which was observed for the RdRP–RDV simulations. The residue-wise fluctuation averaged over the entire simulation length was also calculated for all the residues of RdRP (Fig. S2B†). It was observed that the residues in the range 250–300 and 890–919 fluctuated the most as compared to the other residues. The former residues belong to the interface region which is preceded by the NiRAN domain. The latter residues belong to the thumb subdomain. The fluctuation in the residues were observed in RdRP–Apo as well as RdRP–RDV. However, for RdRP–Apo the RMSF values ranged around 4–5 Å and for RdRP–RDV were slightly lower and around 3–4 Å. Both RMSD and RMSF values inferred that the palm region and thumb region appeared to deviate most from the start model of RdRP. In order to understand the basis of this deviation, principal component analysis was performed on RdRP–Apo and RDV–RdRP simulations. The distribution of the RdRP conformations along the first three principal components was calculated for the RdRP–Apo and RdRP–RDV simulations (Fig. 3). Fig. 3A shows the distribution along the first principal component. It was observed that three distant populations were sampled for the RdRP–Apo and four in case of RdRP–RDV. These populations suggest occurrence of corresponding number of dominant conformations attained by the RdRP in the simulations. Two of the dominant conformations obtained here had overlapping eigen values in both the RdRP–Apo and RdRP–RDV simulations. However, both these states obtained were more populated in case of the apo as compared to the RdRP RDV. The peaks obtained in the RDV-bound system were wide as compared to apo suggesting more variation in the conformations sampled through the simulations. As the variance captured by the principal components decreases with increase in the number of components, the PC2 and PC3 showed the distribution area under the peak reduced, suggesting the capture of conformations with less variance. Although, the RDV-bound system showed a distinct conformation being sampled by the PC2 which was not observed in the population distribution obtained by PC1. The population distribution overlapped for both the simulated systems across PC3. The apo system sampled a single population, whereas RDV-bound system sampled three populations within this distribution. The distribution along the PCs infer that the conformations dominant in the simulations differed in apo and RDV-bound RdRP systems. This may be attributed to the presence of remdesivir in the simulations. In order identify the residues involved in these different dominant conformations obtained the RMSD of all the residues along each of the PCs was calculated. Fig. 4 shows the distribution of residue-wise fluctuation observed from the principal component analysis. Fig. 4A, B and C shows the RMSF for every residue captured by PC1, PC2 and PC3. Fig. 4D–I shows the projections of the variance along the residues of the RdRP. It was observed that the region from residue 880–920 varied significantly as compared the other residues. This region belongs to the thumb region, Fig. 4D and E show the projection of this variation in case of the RdRP–Apo and RdRP–RDV. The porcupine plots provided here, helps to understand the direction along which the conformational variation has occurred. The directionality of the projections indicates that in case of apo these residues of the thumb region tend to move away from the protein, whereas in case of RDV–RdRP they tend to move towards the protein. This region lies at the interface of the thumb domain and finger subdomain, which is known to be the site for template entry. Similarly, this region appeared to fluctuate the most along PC2 and PC3 (Fig. 4E–I). The directionality behavior too was observed to be same as that seen in PC1. Apart, from these residues of the thumb region. The region comprising of the residues 250–270 fluctuated the most in case of the apo simulations along all the three PCs. This region belongs to the interface domain which lies between the NiRaN domain and finger subdomain. However, the fluctuation observed in this region was comparatively lower in the RDV-bound simulations along PC1. The PC2 and PC3 was able to capture similar fluctuations for this region in both apo and RDV-bound system. The residues from the palm subdomain appeared to fluctuate in RDV-bound system along all the three PCs. The fluctuation in this region was captured only in PC3 for the apo simulations. The fluctuations observed in the PC3 suggest that a smaller population was sampled that had the variation in the residues with increased RMSF values. The comparative study using principal component analysis helped to deduce that the presence of remdesivir may have led to significant conformation changes in the RdRP. These changes may have implications in the inhibitory activity of remdesivir. These conformational changes may reflect the role of remdesivir on binding to the RdRP unable the entry of new nucleotides, leading to the inhibition of replication. The simulation and modeling studies reported by Shannon *et al.*, Zhang *et al.* and Aranda *et al.* provide a detailed analysis of with the perspective of remdesivir binding to the RNA template.^31–33^ The principal component analysis performed herein helped in understanding the changes occurring in RdRP on binding to remdesivir. However, the interaction analysis of RdRP–RDV were also performed in order to develop an insight into the mechanism by which the inhibitory effect is brought about by remdesivir.

### RdRP–RDV interaction dynamics

**Fig. 5:**
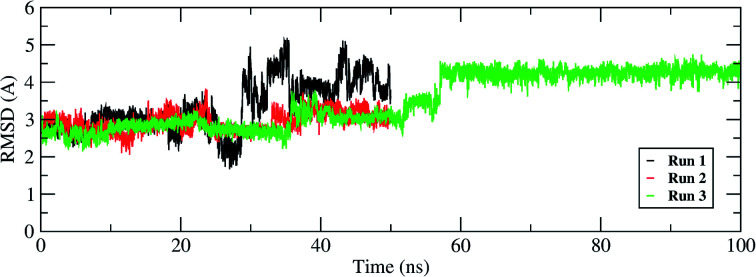
RMSD of the remdesivir bound to RdRP throughout the simulation length for all the three replicates.

**Fig. 6:**
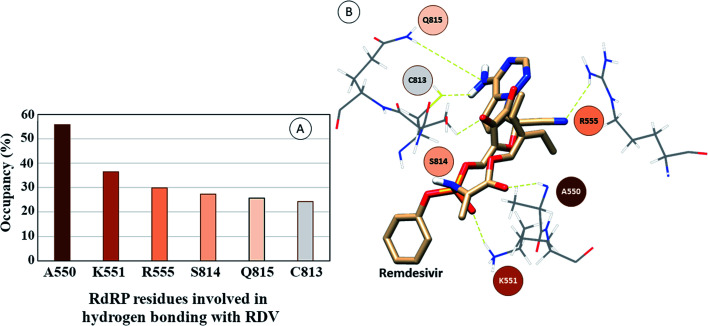
(A) RdRP residues forming hydrogen bonding with RDV with occupancy of more than 20%. (B) Depiction of the location of the residues in the vicinity of remdesivir.

The interactions of remdesivir with RdRP was calculated using different conformational and thermodynamic parameters. The RMSD of the RDV against the direct docked conformation obtained initially was calculated. Fig. 5 shows the RMSD values for RDV molecule obtained throughout the simulation time. Two of the three replicates of the RDV simulations showed fluctuations around 2–3 Å. These two replicates were simulated only for 50 ns. The third replicate was simulated for 100 ns where it was observed that the RMSD stabilized around 4–4.5 Å by the end of the simulations. The last 40 ns of the third replicate showed the RMSD values within this range itself. Few conformations in the range of, 4–4.5 Å were observed in the one of the 50 ns replicate. The nearly constant RMSD values may infer that the RDV attained a stable conformation in the binding pocket of RdRP towards the end of the simulation. After understanding the stability of RDV throughout the simulation, the interactions between the RDV and RdRP were calculated. The interaction analysis included hydrogen bonding, native contacts and free energy of binding. The hydrogen bonding analysis was performed using the PLIP software.^44^ The number of hydrogen bonds formed when RDV is the donor and RdRP is the acceptor and *vice versa* was calculated. The donor–acceptor bond distance of 3.5 Å and the bond angle of 120° were considered as the threshold values for calculation of hydrogen bonds between RDV and RdRP. Fig. 6A shows the number of residues which are involved in formation of hydrogen bonds with RDV for more than 20% of the simulation time were calculated. A cumulative of 200 ns of the RdRP–RDV simulations were considered for this analysis. A total of six RdRP residues were involved in forming hydrogen bond with RDV and having an occupancy more than 20%. The residue ALA 550 formed hydrogen bond with RDV with an occupancy of more than 50%. This was followed by LYS 551 and ARG 555 which showed an occupancy around 38% and 30% respectively. ALA 550, LYS 55, and ARG 55 all the three residues belong to the conserved motif F (Fig. 6B). The remaining three residues CYS 813, SER 814 and GLN 815 were involved in forming hydrogen bonds with RDV with an occupancy ranging between 20–30%. These three residues belong to the conserved motif E (Fig. 6B). These three residues have also been reported to play a role in binding to the primer during the transcription process of SARS-CoV-2.^12^ The hydrogen bonding analysis inferred that the residues belonging to the conserved motifs E and F participate significantly in forming hydrogen bonds with the inhibitor RDV. The depiction of hydrogen bonding at every 10 ns for run1 and run2 has been given in supplementary Fig. S3 and S4† respectively. The same for first 50 ns and last 50 ns of run3 has been given in supplementary Fig. S5 and S6.†

**Fig. 7:**
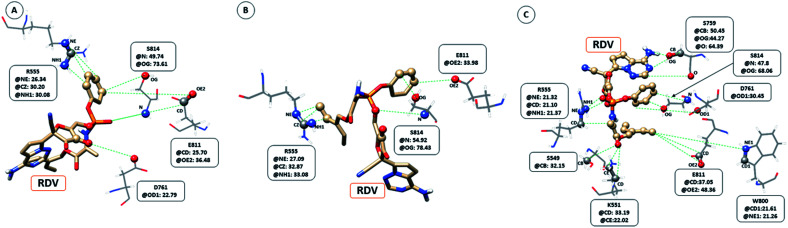
RdRP residues involved in contact formation with RDV and their relative contact strength for run1 (A), run2 (B) and run3 (C) of the RdRP–RDV simulations.

The interaction between RDV and RdRP captured through simulations was further explored by calculating the native contacts formed by the residues of RdRP with RDV throughout the simulations. The *nativecontacts* module of *cpptraj* from AmberTools 17 was used for performing this calculation.^43^ The relative contact strength for contacts between an atom of RDV and an atom of the residues of RdRP was calculated, considering the initial docked state of RDV as the reference. The distance between the atoms of RDV and atoms of the residues of RdRP were calculated and the ones with distance less than 5 Å was considered as a contact. The relative contact strength is a normalized value for every contact such that a value of 100 would mean that the atom was involved in forming the largest number of contacts with the other atoms. Fig. 7 depicts the residues that were involved in forming native contact with a relative contact strength more than 20 in the run1 (50 ns), run2 (50 ns) and run3 (100 ns) of the RdRP–RDV complex simulations. Fig. 7A depicts the results for the run1 of the RDV-RdRP complex. It was observed that four residues were involved in forming contacts with the RDV inhibitor. The atoms represented as spheres in the wire representation of the residues were the ones involved in forming the contacts. The residues ARG 555, ASP 761, GLU 811 and SER 814 were observed to form 3, 1, 2 and 2 number of contacts respectively. However, the relative contact strength for the atoms of SER 814 were comparatively higher than the other three residues. SER 814 belongs to the conserved motif E and is also known to play a role in primer binding. ASP 761 belongs to the conserved motif C and is also known to be one of the catalytic residues of the SDD triplet present in this motif. ARG 555 formed three contacts with relative contact strength better than that observed for ASP 761 belonged to the motif F. Similarly, GLU 811 formed two contacts with a comparatively better contact strength than ASP 761. Fig. 7B depicts the results for the run 2 of the RdRP–RDV complex simulations. In agreement to the results obtained in run 2, ARG 555, GLU 811 and SER 814 were observed to be involved in forming native contacts with RDV. In this simulation too, SER 814 formed strong native contacts with RDV as compared to the other two. Fig. 7C depicts the results for the run 3 of the RdRP–RDV complex simulation which was performed for 100 ns. It was observed that increasing the simulation length helped to capture the involvement of greater number of residues in forming native contacts with the RDV inhibitor. SER 549, LYS 551, ARG 555, SER 759, ASP 761, TRP 800, GLU 811 and SER 814 were observed to form native contacts with RDV with a relative contact strength more than 20. These residues belonged to the conserved motifs F, C and E. Two of the serine residues SER 759 and SER 814 which belonged to the motifs C and E were observed to form strong native contacts with the RDV molecule. SER 759 is also known to be a part of the SDD catalytic site. SER 814 also formed strong native contacts with RDV, which is in well agreement with the cryo-EM structure reported with the PDB ID 7BV2. The other residue from motif E, GLU 811 was also observed to form stronger contacts than what was seen in run1 and run2. The second aspartate of the SDD catalytic site namely, ASP 761 formed strong native contact with RDV molecule. SER 549, LYS 551 and ARG 555 of the conserved motif F were observed to form contacts with the RDV inhibitor. The residues of motif F are known to be involved in interacting with the atoms of RDV. The residue TRP 800 belonged to the palm subdomain was also observed to form contacts with the RDV molecule. The distance-based conformation parameters, hydrogen bond and native contacts were able to identify the residues that are known to interact with RdRP inhibitors.

Investigating the thermodynamic properties explored through the simulations would help in developing a deeper insight into the RDV and RdRP interactions. The molecular mechanics/generalized Born surface area calculations would prove to be useful in determining the free energy of binding between the protein and the ligand molecule.^45^ The free energy of binding was calculated using the *mmpbsa* module of AmberTools 17.^46^ The free energy of binding was calculated using the following equation,

1 ΔΔ*G*_bind_ = Δ*G*_complex_ − (Δ*G*_receptor_ + Δ*G*_ligand_)

2 Δ*G*_complex_ = Δ*H*_complex_ − *T*Δ*S*_complex_ (on similar lines for receptor and ligand)

**Fig. 8:**
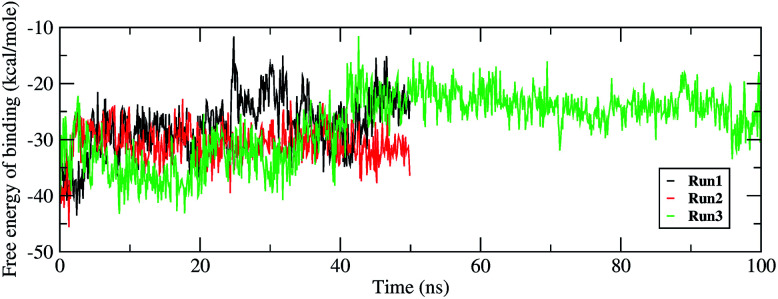
Free energy of binding between RdRP and RDV throughout the simulations for run1, run2 and run3.

**Fig. 9:**
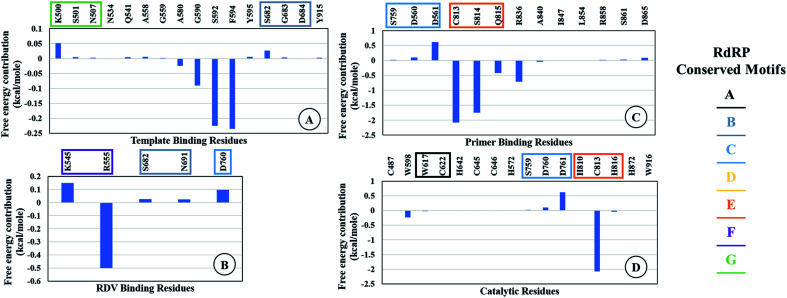
Residue-wise average free energy contribution in binding between RdRP and RDV. (A) Template binding residues, (B) rdz binding residues, (C) primer binding residues, (D) catalytic residues.

The ΔΔ*G*_bind_ is the free energy of binding between RDV and RdRP. The Δ*G*_complex_ is the free energy of the RdRP–RDV complex. The Δ*G*_receptor_ is the free energy of only the RdRP from the RdRP–RDV complex. The Δ*G*_ligand_ is the free energy of only the RDV from the RdRP–RDV complex. Δ*H* is the internal energy which accounts for van der Waals, electrostatics and solvation effect and *T*Δ*S* is the entropy, that measures the degree of disorderness of a system. The calculation of *T*Δ*S* is very time consuming, hence, only the Δ*H* was calculated for all the simulations. Fig. 8 depicts the free energy of binding between RDV and RdRP throughout the simulations for run1, run2 and run3. It was observed that the run1 and run2 have fluctuating values for the free energy of binding similar to what was observed for RMSD of the RDV molecule. Run3 simulation of the RdRP–RDV complex shows stable free energy of binding in the last 40 ns of the simulations. The free energy value was observed to fluctuate with 5 kcal mol^−1^ with most of the conformations having the value around −25 kcal mol^−1^. Owing this observation, the residue-wise free energy contribution in binding was calculated for all the three runs. The average free energy contribution in binding between RDV and RdRP has been shown in Fig. 9. Fig. 9A shows the free energy contribution for the residues known to play a role in the template binding. The residues ALA 580, GLY 590, SER 592 and PHE 594 were observed to contribute in the free energy of binding, with PHE 594 showing the maximum contribution compared to others. Except for ALA 580, the remaining three residues belong to the palm subdomain of RdRP. Fig. 9B shows the free energy contribution for the residues known to involved in binding to the RDV inhibitor. ARG 555, a part of the conserved motif F showed maximum contribution in binding to the RDV inhibitor. This observation was in well agreement with the distance-based conformational analysis, where ARG 555 was observed to be actively involved in forming hydrogen bond and native contacts with the RDV inhibitor. Fig. 9C depicts the free energy contribution for the residues known to be involved in the primer binding. The CSQ triplet corresponding to CYS 813, SER 814 and GLN 815 was observed to contribute in the free energy of binding between RDV and RdRP. The hydrogen bond analysis also showed that these residues were significantly involved in forming hydrogen bonds with RDV. Apart from these three ARG 836 which belongs to the thumb subdomain was also observed to contribute in the free energy of binding between RDV and RdRP. Amongst, the residues involved in the catalytic site of the RdRP, TRP 598 and CYS 813 were observed to contribute to the free energy of binding (Fig. 9D). The calculation of the thermodynamic properties in terms of MMGBSA free energy of binding helped in identifying few more residues from the palm subdomain namely, GLY 590, SER 592 and PHE 594 that may prove important in inhibitor interactions.

### Ensemble docking of remdesivir

In addition to the molecular dynamics simulations of RdRP–RDV complex obtained from the direct docking of the RDV on the RdRP model, an ensemble docking was also performed. Ensemble results in generation of conformations that explore the binding pocket of the receptor molecule and enable better binding of ligand molecules. One of the previous works, on the ensemble docking approach to screen drug molecules against the 3C-like protease of SARS-CoV-2 had enabled in finding more relevant drugs that show strong binding against the drug target.^47^ An ensemble of RdRP was generated by clustering the RdRP–Apo simulation data. A total of 5 clusters were obtained, the cluster centers of these clusters were considered for ensemble docking of RDV. The remdesivir was docked on to these ensemble representatives of RdRP. The interaction energy was calculated between the RDV and RdRP using the PRODIGY-LIG server.^48^Table 1 shows the interaction energies obtained for every ensemble docked structure and the RMSD of the corresponding RdRP ensemble representative against the RdRP of PDB ID: 7BV2.^12^

**Table 1:** Interaction energies obtained for RDV docked on to RdRP ensemble representatives and their RMSD against PDB ID: 7BV2

RdRP ensemble representative	Interaction energy (kcal mol^−1^)	RMSD against PDB ID 7BV2 (Å)
1	−8.6	2.032
2	−8.5	2.164
3	−8.1	2.014
4	−8.5	2.911
5	−8.8	1.681

The interaction energies obtained for RdRP–RDV ensemble docked structures ranged within −8.8 to −8.1 kcal mol^−1^. These observations suggest that all the ensemble docked conformations showed similar binding affinities amongst one another. However, on comparing the RMSD values of these ensemble representatives against the RdRP of 7BV2 revealed that the representative with the least RMSD of 1.681 Å appeared to predict the best interaction energy. This interaction energy indicates the presence of strong interactions between the RDV and RdRP.

**Fig. 10:**
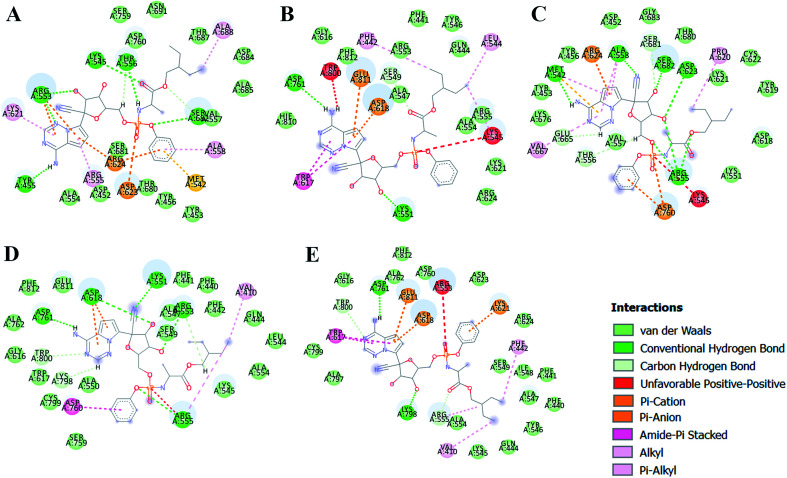
RDV interactions with the residues of RdRP in each of the five ensemble representative structures of RdRP (A–E).

The different types of interactions made by RDV with each of these ensemble representatives has been shown in the Fig. 10.^49^ The inhibitor was observed to form van der Waals, hydrogen bond and π-anion/cation interactions with each of the ensemble representatives of RdRP. The residues namely, TYR 451, LYS 540, MET 542, ARG 548, LYS 551, ARG 553, ARG 555, ALA 558, ASP 618, SER 674, ASP 761, and GLU 811 were observed to interact with RDV. ARG 555 was observed to be involved in hydrogen bonding in all the five ensemble docked structures. The aspartate residues of the SDD signature catalytic region were also observed to form π-cationic/anionic interactions with RDV in two of the ensemble-docked conformers. It was observed that the residues known to be crucial in interacting with the inhibitor from the literature and obtained through the simulations matched the ones seen in ensemble docking. Hence, ensemble docking results were in well agreement with the observations obtained from the simulations of RdRP–RDV complex.

## Conclusion

Remdesivir, an inhibitor designed to abrogate the activity of the most conserved viral protein RNA dependent RNA polymerase was studied using molecular dynamics simulations and ensemble docking approaches. The comparative study performed for the apo form and remdesivir-bound form of RNA dependent RNA polymerase revealed conformational dynamics contrasting to one another. Principal component analysis inferred that the movement of the thumb subdomain near the template entry site and the interface region between the NiRAN domain and finger subdomain were completely opposite in apo and remdesivir-bound RNA dependent RNA polymerase. The movement observed for remdesivir-bound system suggested blocking of the template entry site, whereas for apo system it inferred opening of the template entry site. The strong binding of remdesivir to primer binding inferred that it is making them unavailable for binding to the natural nucleotides. The remdesivir-bound simulations revealed strong hydrogen bonding and native contacts between the residues belonging to the conserved motifs with the remdesivir molecule. The thermodynamic parameters studied to understand the interaction of remdesivir supported the observations obtained through conformational analysis. The residues SER 549, LYS 551, ARG 555 (motif F), SER 759, ASP 760, ASP 761 (motif C), CYS 813, SER 814 and GLN 815 (motif E) belonging to the conserved motifs pose at the crucial residues that need to be targeted for inhibitor design. The ensemble docking approach proved to support the results obtained through simulations. Most of the crucial residues matched in both the cases. The results presented in this article were also in well agreement with the recently reported cryoEM structures on remdesivir bound RNA dependent RNA polymerase complexes. The *in silico* approaches used in the current work possess significant scope in designing inhibitors by mapping their functional groups on to the interacting residues of the conserved regions of the drug target. The work presented in this article has been published as a preprint earlier.^50^

## Conflicts of interest

There are no conflicts of interest to declare.

## Supplementary Material

RA-010-D0RA04743K-s001

## References

[cit1] Andersen K. G., Rambaut A., Lipkin W. I., Holmes E. C., Garry R. F. (2020). Nat. Med..

[cit2] Alanagreh L. A., Alzoughool F., Atoum M. (2020). Pathogens.

[cit3] Kim D., Lee J. Y., Yang J. S., Kim J. W., Kim V. N., Chang H. (2020). Cell.

[cit4] Wang H., Li X., Li T., Zhang S., Wang L., Wu X., Liu J. (2020). Eur. J. Clin. Microbiol. Infect. Dis..

[cit5] Gaurav A. and Al-Nema M., in Book Viral Polymerases: Structures, Functions and Roles as Antiviral Drug Targets, ed. S. P. Gupta, Academic Press, India, 2019, vol. 10, pp. 271–300, 10.1016/B978-0-12-815422-9.00010-3

[cit6] Jia H., Gong P. (2019). Front. Microbiol..

[cit7] Shafique L., Ihsan A., Liu Q. (2020). Pathogens.

[cit8] Srinivasan S., Cui H., Gao Z., Liu M., Lu S., Mkandawire W., Narykov O., Sun M., Korkin D. (2020). Viruses.

[cit9] Xu X., Liu Y., Weiss S., Arnold E., Sarafianos S. G., Ding J. (2003). Nucleic Acids Res..

[cit10] Xu J., Zhao S., Teng T., Abdalla A. E., Zhu W., Xie L., Wang Y., Guo X. (2020). Viruses.

[cit11] Gordon D. E., Jang G. M., Bouhaddou M., Xu J., Obernier K., O'Meara M. L., Guo J. Z., Swaney D. L., Tummino T. A., Hüttenhain R., Kaake R. M., Richards A. L., Tutuncuoglu B., Foussard H., Batra J., Haas K K., Modak M., Kim M., Haas P., Polacco B. J., Braberg H., Fabius J. M., Eckhardt M., Soucheray M., Bennett M. J., Cakir M., McGregor M. J., Li Q., Naing Z. Z. C., Zhou Y., Peng S., Kirby I. T., Melnyk J. E., Chorba J. S., Lou K., Dai S. A., Shen W., Shi Y., Zhang Z., Barrio-Hernandez I., Memon D., Hernandez-Armenta C., Mathy C. J. P., Perica T., Pilla K. B., Ganesan S. J., Saltzberg D. J., Ramachandran R., Liu X., Rosenthal S. B., Calviello L., Venkataramanan S., Lin Y., Wankowicz S. A., Bohn M., Trenker R., Young J. M., Cavero D., Hiatt J., Roth T., Rathore U., Subramanian A., Noack J., Hubert M., Roesch F., Vallet T., Meyer B., White K. M., Miorin L., Agard D., Emerman M., Ruggero D., García-Sastre A., Jura N., von Zastrow M., Taunton J., Schwartz O., Vignuzzi M., d'Enfert C., Mukherjee S., Jacobson M., Malik H. S., Fujimori D. G., Ideker T., Craik C. S., Floor S., Fraser J. S., Gross J J., Sali A., Kortemme T., Beltrao P., Shokat K., Shoichet B. K., Krogan N. J. (2020). Nature.

[cit12] Gao Y., Yan L., Huang Y., Liu F., Zhao Y., Cao L., Wang T., Sun Q., Ming Z., Zhang L., Ge J. (2020). Science.

[cit13] Li G., De Clercq E. (2020). Nat. Rev. Drug Discov..

[cit14] Buonaguro L., Tagliamonte M., Tornesello M. L., Buonaguro F. M. (2020). J. Transl. Med..

[cit15] Elfiky A. A. (2020). J. Biomol. Struct. Dyn..

[cit16] Elfiky A. A. (2020). Life Sci..

[cit17] Sayad B., Sobhani M., Khodarahmi R. (2020). Arch. Med. Res..

[cit18] McKee D. L., Sternberg A., Stange U., Laufer S., Naujokat C. (2020). Pharmacol. Res..

[cit19] Wu C., Liu Y., Yang Y., Zhang P., Zhong W., Wang Y., Wang Q., Xu Y., Li M., Li X., Zheng M. (2020). Acta Pharm. Sin. B.

[cit20] YAVUZ S., Ünal S. (2020). Turk. J. Med. Sci..

[cit21] Tchesnokov E. P., Feng J. Y., Porter D. P., Götte M. (2019). Viruses.

[cit22] Seigel D., Hui H. C., Doerffler E., Clarke M. O., Chun K., Zhang L., Neville S., Carra E., Lew W., Ross B., Wang Q., Wolfe L., Jordan R., Soloveva V., Knox J., Perry J., Perron M., Stray K. M., Barauskas O., Feng J. Y., Xu Y., Lee G., Rheingold A. L., Ray A. S., Bannister R., Strickley R., Swaminathan S., Lee W. A., Bavari S., Cihlar T., Lo M. K., Warren T. K., Mackman R. L. (2017). J. Med. Chem..

[cit23] Gordon C. J., Tchesnokov E. P., Feng J. Y., Porter D. P., Götte M. (2020). J. Biol. Chem..

[cit24] Gordon C. J., Tchesnokov E. P., Woolner E., Perry J. K., Feng J. Y., Porter D. P., Götte M. (2020). J. Biol. Chem..

[cit25] Huang J., Song W., Huang H., Sun Q. (2020). Clin. Med..

[cit26] Kaul D. (2020). Current Medicine Research and Practice.

[cit27] Khan Z., Karataş Y., Rahman H. (2020). Adv. Ther..

[cit28] Ledford H. (2020). Nature.

[cit29] Ko W. C., Rolain J. M., Lee N. Y., Chen P. L., Huang C. T., Lee P. I., Hsueh P. R. (2020). Int. J. Antimicrob. Agents.

[cit30] Yin W., Mao C., Luan X., Shen D. D., Shen Q., Su H., Wang X., Zhou F., Zhao W., Gao M., Chang S., Xie Y. C., Tian G., Jiang H. W., Tao S. C., Shen J., Jiang Y., Jiang H., Xu Y., Zhang S., Zhang Y., Xu H. E. (2020). Science.

[cit31] Shannon A., Le N. T., Selisko B., Eydoux C., Alvarez K., Guillemot J. C., Decroly E., Peersen O., Ferron F., Canard B. (2020). Antiviral Res..

[cit32] Zhang L., Zhou R. (2020). J. Phys. Chem. B.

[cit33] Juan A., Modesto O. (2020). bioRxiv.

[cit34] Kirchdoerfer R. N., Ward A. B. (2019). Nat. Commun..

[cit35] Bienert S., Waterhouse A., de Beer T. A., Tauriello G., Studer G., Bordoli L., Schwede T. (2017). Nucleic Acids Res..

[cit36] Pettersen E. F., Goddard T. D., Huang C. C., Couch G. S., Greenblatt D. M., Meng E. C., Ferrin T. E. (2004). J. Comput. Chem..

[cit37] Allen W. J., Balius T. E., Mukherjee S., Brozell S. R., Moustakas D. T., Lang P. T., Case D. A., Kuntz I. D., Rizzo R. C. (2015). J. Comput. Chem..

[cit38] Case D. A. , Betz R. M., Cerutti D. S., Cheatham III T. E., Darden T. A., Duke R. E., Giese T. J., Gohlke H., Goetz A. W., Homeyer N., Izadi S., Janowski P., Kaus J., Kovalenko A., Lee T. S., LeGrand S., Li P., Lin C., Luchko T., Luo R., Madej B., Mermelstein D., Merz K. M., Monard G., Nguyen H., Nguyen H. T., Omelyan I., Onufriev A., Roe D. R., Roitberg A., Sagui C., Simmerling C. L., Botello-Smith W. M., Swails J., Walker R. C., Wang J., Wolf R. M., Wu X., Xiao L. and Kollman P. A., AMBER 2016, University of California, San Francisco. 2016

[cit39] Wang J., Wang W., Kollman P. A., Case D. A. (2006). J. Mol. Graphics Modell..

[cit40] Wang W. R., Wolf R. J., Caldwell J. W., Kollman P. A., Case D. A. (2004). J. Comput. Chem..

[cit41] Peters M. B., Yang Y., Wang B., Fusti-Molnar L., Weaver M. N., Merz Jr K. M. (2010). J. Chem. Theory Comput..

[cit42] Ester M. , Kriegel H. P., Sander J. and Xu X., Proceedings of the Second International Conference on Knowledge Discovery and Data Mining (KDD-96), 1996, pp. 226–231

[cit43] Roe D. R., Cheatham III T. E. (2018). J. Comput. Chem..

[cit44] Sebastian S., Sven S., Joachim H. V., Adasme Melissa F., Schroeder M. (2015). Nucleic Acids Res..

[cit45] Genheden S., Ryde U. (2015). Expert Opin. Drug Discovery.

[cit46] Miller Jr B. R., McGee D. T., Swails J. M., Homeyer N., Gohlke H., Roitberg J. (2012). J. Chem. Theory Comput..

[cit47] Koulgi S., Jani V., Uppuladinne M., Sonavane U., Nath A. K., Darbari H., Joshi R. (2020). J. Biol. Struc. Dyn..

[cit48] Vangone A., Schaarschmidt J., Koukos P., Geng C., Citro N., Trellet M. E., Xue L. C., Bonvin A. M. (2019). Bioinformatics.

[cit49] Dassault Systèmes BIOVIA, BIOVIA Workbook, Release 2019; BIOVIA DS Visualizer, Release 2019, San Diego: Dassault Systèmes, [2019]

[cit50] Koulgi S., Jani V., Uppuladinne M., Sonavane U., Joshi R. (2020). ChemRxiv.

