# Supplementary material for: Remdesivir-bound and ligand-free simulations reveal the probable mechanism of inhibiting the RNA dependent RNA polymerase of severe acute respiratory syndrome coronavirus 2
Source: RSC Adv. 2020 Jul 17;10(45):26792–803. doi: 10.1039/d0ra04743k (PMC9055499; doi:10.1039/d0ra04743k)
Supplement: RA-010-D0RA04743K-s001 [file RA-010-D0RA04743K-s001.pdf]

Electronic Supplementary Material (ESI) for RSC Advances.
This journal is © The Royal Society of Chemistry 2020

Supplementary data for “Remdesivir-bound and ligand-free simulations reveal the probable mechanism of inhibiting the RNA dependent RNA polymerase of Severe Acute

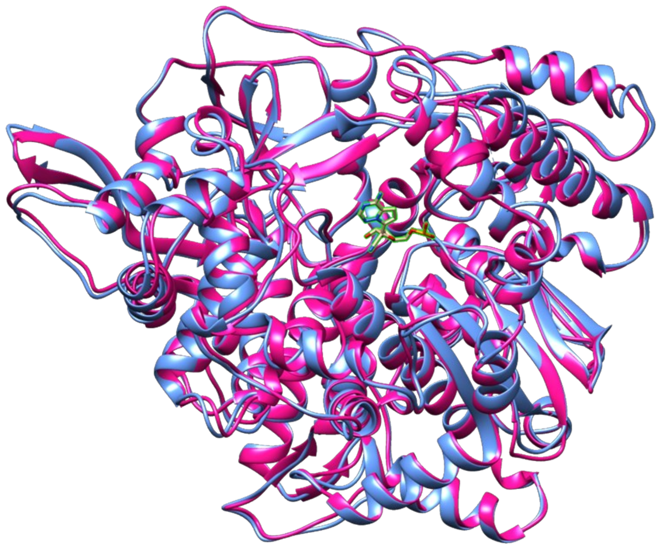

Supplementary Figure S1: Superimposition of the RdRP model (pink) and 7BV2 (blue)

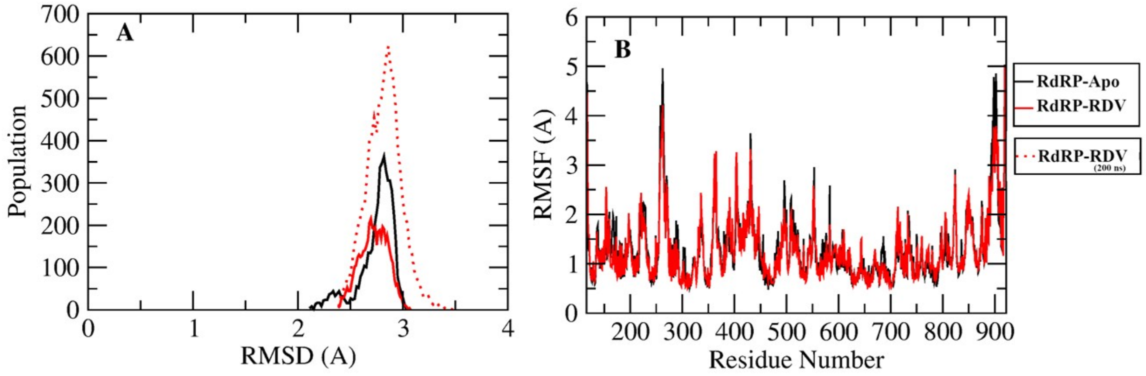

Supplementary Figure S2: A: RMSD of the polymerase domain against the RdRP model.
B: RMSF of the residues of polymerase domain throughout the simulation length

Respiratory Syndrome Coronavirus 2”

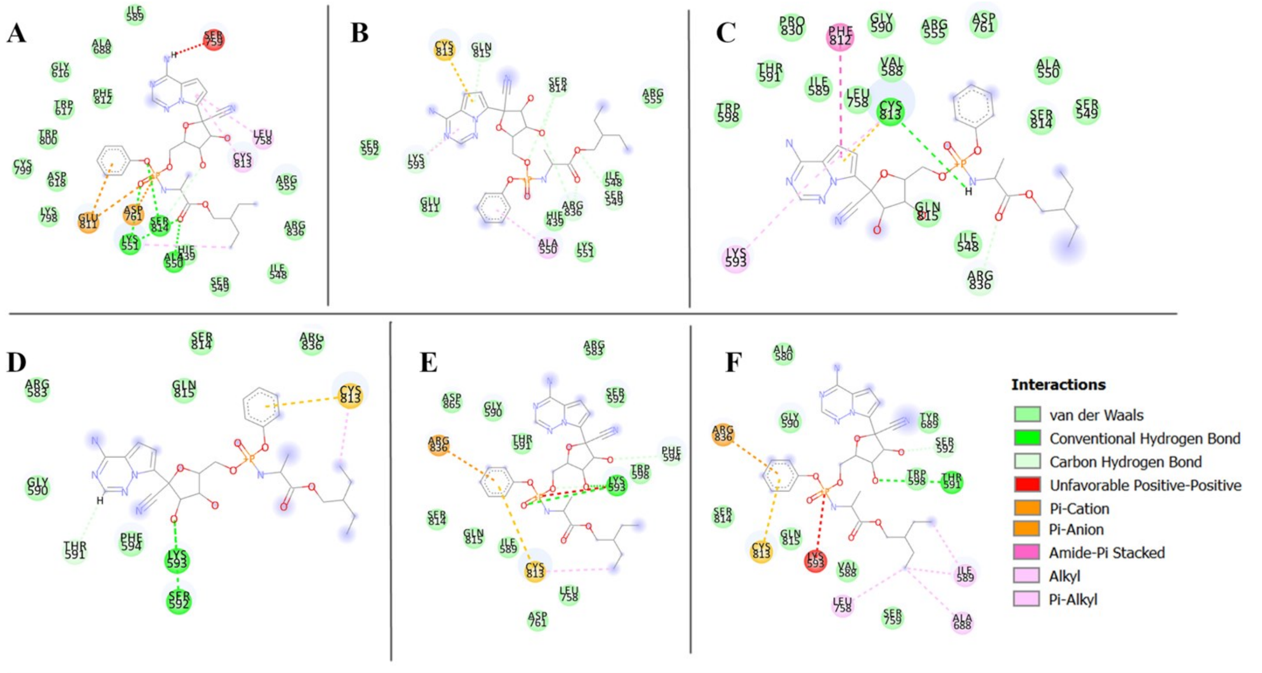

Figure S3: Interactions between RDV and RdRP for run1 of the RdRP-RDV system simulations. The interactions at (A) 0thns, (B) 10thns, (C) 20thns, (D) 30thns, (E) 40thns and (F) 50thns

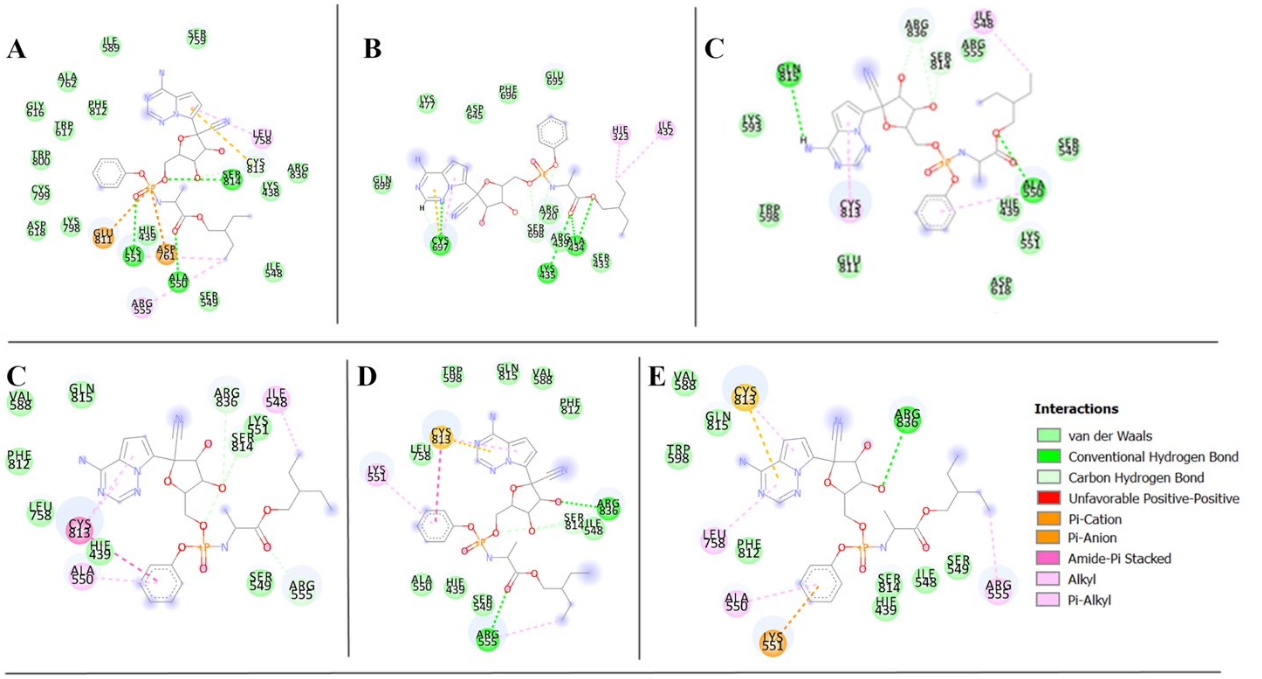

Figure S4: Interactions between RDV and RdRP for run2 of the RdRP-RDV system simulations. The interactions at (A) 0thns, (B) 10thns, (C) 20thns, (D) 30thns, (E) 40thns and (F) 50thns

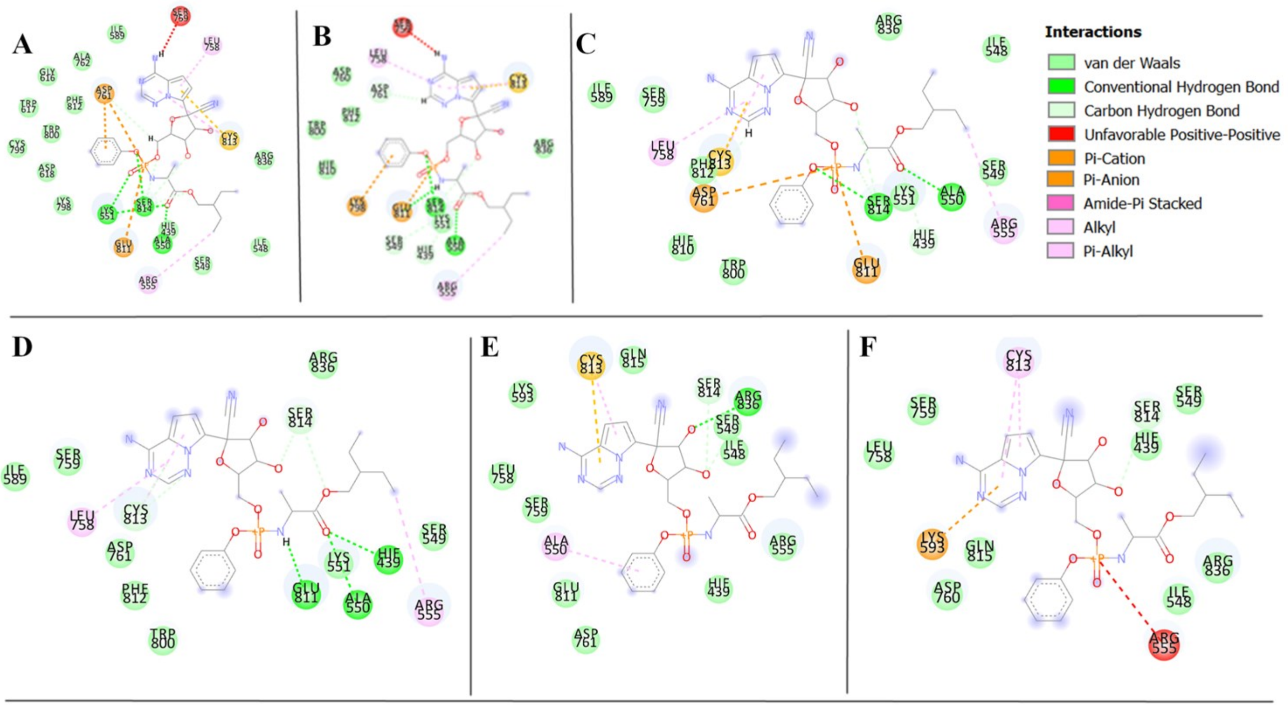

Figure S5: Interactions between RDV and RdRP for run3 of the RdRP-RDV system simulations. The interactions at (A) 0thns, (B) 10thns, (C) 20thns, (D) 30thns, (E) 40thns and (F) 50thns

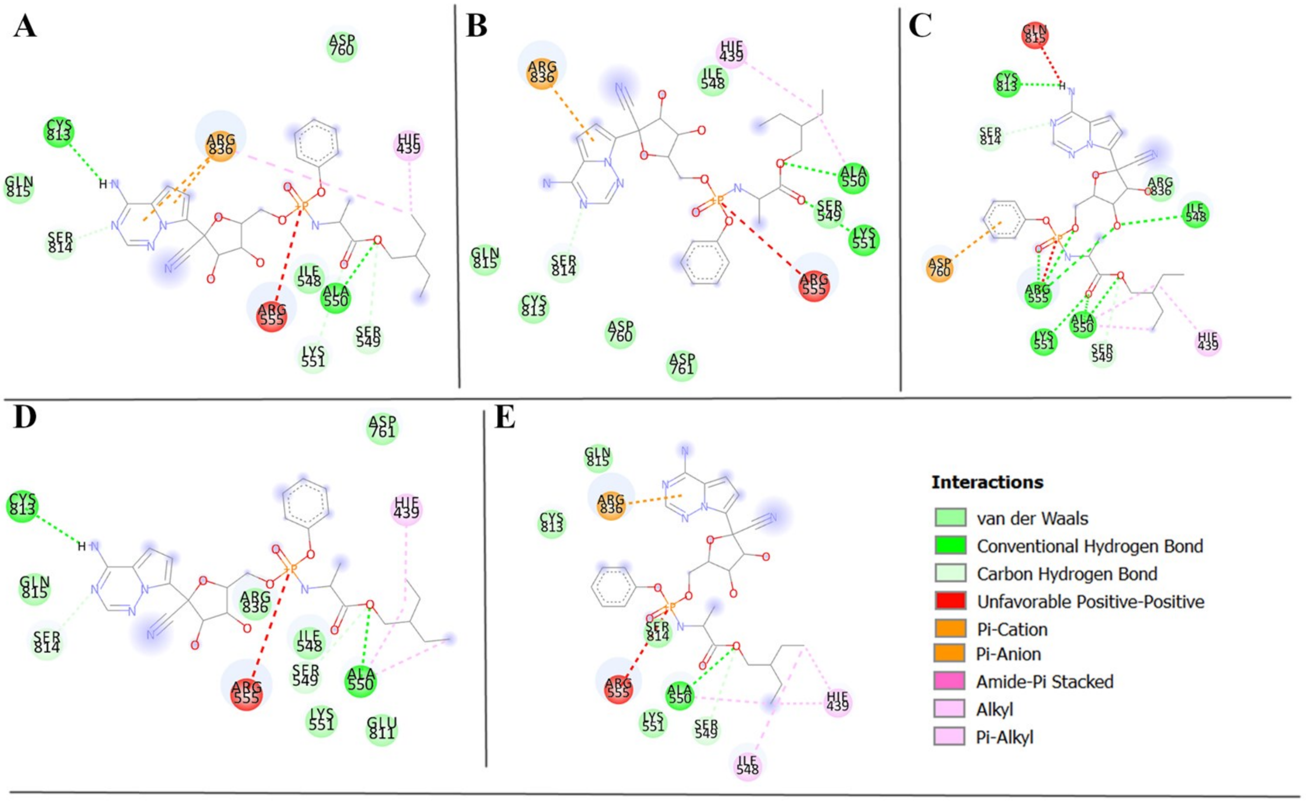

Figure S6: Interactions between RDV and RdRP for run3 of the RdRP-RDV system simulations. The interactions at (A) 60thns, (B) 70thns, (C) 80thns, (D) 90thns and (E) 100thns
